# Exploring the impact of dietary theobromine on endometriosis risk: Evidence from Mendelian randomization and NHANES data

**DOI:** 10.1097/MD.0000000000045989

**Published:** 2025-11-21

**Authors:** Yingqin Huang, Feng Liang, Baoli Xie, Dongmei Huang

**Affiliations:** a Department of Reproductive Medicine, Reproductive Medicine Center, Maternity and Child Health Care of Guangxi Zhuang Autonomous Region, Nanning, Guangxi, China; b Gynecology Department, The Reproductive Hospital of Guangxi Zhuang Autonomous Region, Nanning, China; c Gynecology Department, The First People’s Hospital of Nanning, Nanning, China.

**Keywords:** dietary intake, endometriosis, Mendelian randomization, NHANES, theobromine

## Abstract

Endometriosis is a chronic inflammatory estrogen-dependent inflammatory disorder. Theobromine has been implicated in diverse health benefits, including anti-inflammatory and vasodilatory properties. However, the precise association between theobromine and endometriosis remains largely undetermined. To address this gap, this study sought to explore the causal relationship between theobromine exposure and the risk of endometriosis by leveraging Mendelian randomization (MR) and data from the National Health and Nutrition Examination Survey (NHANES). This study included 244 participants from the NHANES (1999–2004). Multivariable logistic regression (adjusted for age, body mass index, smoking, alcohol intake, and energy intake) assessed associations between dietary theobromine intake and endometriosis diagnosis age. Two-sample MR (16,588 cases/1,11,583 controls) used 12 theobromine-associated single-nucleotide polymorphisms (*P* < 5 × 10⁻⁸) as instruments, with inverse variance weighted analysis. MR assumptions were verified via *F*-statistics (>10), heterogeneity (Cochran’s *Q P* > .05), and pleiotropy (MR-Egger *P* > .05). In the observational study (NHANES), each 1 μg/d increment in dietary theobromine intake was associated with a 1.49-year delay in endometriosis diagnosis age (β = 1.49, 95% CI: 0.02–2.95, *P* = .048) after multivariable adjustment. In the MR analysis, the inverse variance weighted method showed a significant causal effect estimate of β = −0.1057 (95% CI: −0.2098 to −0.0016, *P* = .045) for the effect of theobromine on endometriosis risk. Sensitivity analyses confirmed robustness against pleiotropy and heterogeneity. This study suggests a potential causal relationship between dietary theobromine intake and a modestly reduced risk of endometriosis, providing preliminary insights into the potential protective effects of theobromine against the development of this condition. However, further investigations are necessary to validate these findings and to understand the underlying biological mechanisms.

## 1. Introduction

Endometriosis is a chronic, prevalent inflammatory disorder defined by the ectopic growth of endometrial-like tissue outside the uterine cavity, commonly involving pelvic organs and tissues. It affects approximately 176 million women globally, with 5% to 10% of women of reproductive age manifesting symptoms such as pelvic pain and infertility.^[1]^ Notably, women with dysmenorrhea exhibit a 40% to 60% higher risk of developing endometriosis, whereas those with subfertility face a 21% to 47% increased risk, and individuals with chronic pelvic pain show an 81% to 87% elevated risk.^[2]^ Beyond its clinical burden, healthcare costs for women with endometriosis are over twice as high as those of unaffected women.^[3]^ Given its significant global prevalence and considerable impact on healthcare expenditure and quality of life,^[4]^ endometriosis is recognized as a major public health concern.^[5]^ Against this backdrop, recent years have witnessed increasing attention directed to nutrition, explored both as a modifiable risk factor and a potential therapeutic strategy for managing the condition.^[6–8]^ Theobromine, a methylxanthine compound predominantly present in cocoa and chocolate, has been reported to exert diverse pharmacological activities.^[9]^ For instance, it enhances the chemosensitivity of colorectal cancer cells, promotes apoptosis, and simultaneously inhibits DNA synthesis and cell proliferation.^[10]^ Additionally, in glioblastoma cells, theobromine activates pro-apoptotic signaling cascades while suppressing the proliferative and antiapoptotic Akt/mTOR and ERK pathways.^[11]^ Beyond these oncological effects, theobromine demonstrates marked anti-inflammatory and antioxidant properties, facilitating the immune system’s production of anti-inflammatory mediators. This immunomodulatory capacity contributes to the preservation of epithelial barriers and mitigates the risk of inflammatory disorders such as dermatitis, intestinal inflammation, and osteoarthritis.^[12]^ Owing to its natural derivation and comparatively low side-effect profile relative to other methylxanthines, theobromine has garnered substantial attention from both the general public and the scientific community. To the best of our knowledge, no prior studies have specifically examined the association between dietary theobromine intake and the age at endometriosis diagnosis. To address this critical knowledge gap, we investigated the relationship between dietary theobromine consumption and the age of endometriosis diagnosis using data from the National Health and Nutrition Examination Survey (NHANES). Furthermore, we performed a 2-sample Mendelian randomization (MR) analysis to investigate the potential causal relationship between theobromine levels and endometriosis. This integrated analytical approach enables a comprehensive assessment of both the observational association and potential causality between theobromine and endometriosis.

## 2. Materials and methods

### 2.1. Data sources of Mendelian randomization analysis and cross-sectional study

Data were derived from the NHANES, administered by the National Center for Health Statistics (NCHS). Given that questions regarding the age at endometriosis diagnosis were exclusively included in NHANES cycles from 1999 to 2004, our analysis was restricted to data from these years. Data collection involved physical examinations and structured interviews, utilizing a nationally representative, stratified sampling design across 3 consecutive 2-year NHANES cycles during this period. The study protocol was approved by the NCHS Ethics Review Committee, and all participants provided written informed consent prior to enrollment.

For the MR analysis, we employed a 2-sample design in conjunction with publicly available summary statistics from large-scale genome-wide association studies (GWASs). To reduce confounding related to population ancestry, we selected European ancestry-based instrumental variables (IVs) derived from a GWAS of fasting serum theobromine concentrations measured by liquid chromatography-mass spectrometry (LC–MS) in 8137 participants.^[13]^ Endometriosis GWAS summary statistics were retrieved from the Finngen database, comprising 1,28,171 European adult participants (16,588 cases and 1,11,583 controls). The dataset is accessible at: https://storage.googleapis.com/finngen-public-data-r10/summary_stats/finngen_R10_N14_ENDOMETRIOSIS.gz.

### 2.2. Mendelian randomization analysis design and genetic instruments extraction

**Figure 1.**
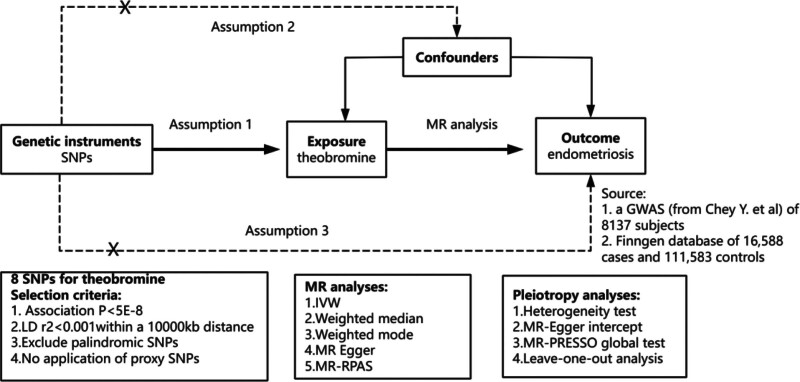
Principles of Mendelian randomization and assumptions. Assumption 1: exposure is robustly associated with genetic variants; assumption 2: confounders are not associated with genetic variants; and assumption 3: genetic variants should influence the outcomes only mediated by the exposure of interest. GWAS = genome-wide association study, IQR = interquartile range, IVW = inverse variance weighted, LD = linkage disequilibrium, MR = Mendelian randomization, MR-PRESSSO = Mendelian Randomization Pleiotropy RESidual Sum and Outlier, MR-RPAS = Mendelian Randomization - Robust and Powerful Association Test, SNP = single-nucleotide polymorphism.

MR analysis offers inherent advantages over traditional observational methods due to the random allocation of genetic variants during gamete formation and their independence from environmental confounding. This design reduces susceptibility to bias from reverse causation and confounding factors. In this study, we utilized MR analysis to identify single-nucleotide polymorphisms (SNPs) associated with both endometriosis and theobromine metabolites. These SNPs were then combined to evaluate the relationship between endometriosis risk and theobromine metabolite levels. To ensure reliable causal inference, the genetic variants selected as IVs in MR analyses must satisfy the following 3 core criteria (Fig. 1) – relevance: the genetic variants must be robustly associated with theobromine metabolite levels; independence: they must not be independent of confounding factors that influence both theobromine metabolism and endometriosis risk; and exclusion restriction: they should influence endometriosis risk exclusively through the metabolites, with no direct effect on endometriosis via alternative biological pathways. SNPs associated with theobromine were selected as candidate genetic instruments using a genome-wide significance threshold (*P* < 5 × 10⁻⁸). With the European 1000 Genomes Project dataset serving as the linkage disequilibrium reference panel, we retained only SNPs with a linkage disequilibrium coefficient (*r*²) < 0.001 to minimize multicollinearity. SNPs demonstrating genome-wide association with endometriosis (using a significance threshold of *P* < 5 × 10⁻⁸) were excluded to avoid pleiotropy. Additionally, SNPs with insufficient *F*-statistics (a metric indicating potential weak instrument bias) were removed from the analysis to ensure robust causal inference.

We selected SNPs explaining a substantial proportion of phenotypic variation in theobromine levels based on 3 key criteria: genome-wide significance (*P* < 5 × 10⁻⁸), clumping analysis to ensure independence (using the European 1000 Genomes reference panel), and *F*-statistics > 10 to validate their strength as instruments. The NOME assumption for MR-Egger was indirectly supported by excluding weak instruments (*F*-statistic < 10). Notably, all retained SNPs exhibited *F*-statistics exceeding 10, a threshold indicating minimal risk of weak instrument bias influencing the causal effect estimates. This rigorous selection framework ensures that the SNPs employed as IVs in our analysis are robust, independent, and strongly associated with theobromine levels. By adhering to these criteria, we enhance the validity of causal inferences derived from the MR analysis. This resulted in 8 independent SNPs. The proportion of phenotypic variance in theobromine levels explained by the 8 instrumental SNPs was calculated as 0.85% (range: 0.12–1.98% per SNP). This estimate was derived using the formula: *R*^2^ = 2 × EAF × (1 − EAF) × β_2_ × EAF/[2 × EAF × (1 − EAF) × β_2_ + se_2_ × N × EAF × (1 − EAF)], where β = effect size, se = standard error, EAF = effect allele frequency, and N = sample size (8137).^[14]^ Although the total *R*^2^ is modest, all SNPs had *F*-statistics > 10 (mean = 28.6), confirming sufficient instrument strength to avoid weak instrument bias.

### 2.3. Cross‑sectional study design and participant screening

The age at endometriosis diagnosis was ascertained from responses to a dedicated question in the reproductive health questionnaire: “How old were you when you were first told you had endometriosis?” Participants who reported a specific age at diagnosis were classified as patients. Given that the questionnaire targeted individuals aged 20 to 54, our study population was restricted to this age group.

In accordance with the study design, participants were randomly assigned to attend data collection appointments scheduled for morning, afternoon, or evening. To assess dietary theobromine and nutrient intake, we utilized the USDA Survey Nutrients Database and the University of Texas Food Intake Analysis System, with explicit exclusion of data from pharmaceuticals and dietary supplements. Two 24-hour dietary recall interviews were conducted: 1 in-person at the Mobile Examination Center (MEC) and the second via telephone 3 to 10 days later. The 24-hour recall method – recognized as a standard approach in large-scale nutritional surveys – was selected for this analysis.^[15]^ Dietary theobromine intake was specifically assessed within the NHANES dietary survey framework, through its “What We Eat in America” survey. This component was implemented at the MEC, where professional interviewers administered the 24-hour recall. The NHANES computer-assisted dietary interview (CADI) system was employed to systematically capture participants’ food and beverage consumption data for the 24-hour period preceding the interview. Covariates included in the analysis comprised age, race/ethnicity, education level, family income, smoking status, physical activity, body mass index (BMI), calorie intake, total fat consumption, carbohydrate consumption, and dietary supplement use.^[16–18]^ Race/ethnicity was categorized into 4 groups: non-Hispanic White, non-Hispanic Black, Mexican American, and other races. Education levels were defined as ordinal categories: <9 years of schooling, 9 to 12 years, and >12 years. Family income was assessed using the poverty income ratio (PIR), as reported by the US Department of Agriculture.^[19]^ Poverty income ratio values were stratified into low, medium, and high incomes tiers based on the range 1.3 to 3.5. Smoking status was categorized as either smokers or never smokers. Physical activity was classified into 3 categories: no physical activity, moderate activity (at least 10 minutes of movement causing light perspiration or a mild to moderate increase in respiration or heart rate in the past 30 days), and vigorous activity (at least 10 minutes of activity causing profuse sweating or a substantial increase in heart rate in the last 30 days). Nutritional data – encompassing calorie and macronutrient intake – were collected via a dietary recall interview administered prior to the MEC interview. Concurrently, data on medications and dietary supplements utilized in the preceding month were recorded.

### 2.4. Statistical analysis

The analysis strictly followed the complex sampling designs and weighting protocols specified by NHANES.^[20]^ Dietary sample weights were applied in weighted analysis: NHANES 1999–2000 and 2001–2002 data were analyzed using the 4-year dietary weight (WTDR4YR), while NHANES 2003–2004 data utilized the day-1 dietary sample weight (WTDRD1). All statistical procedures incorporated these sample weights. Continuous variables were reported as means ± standard deviation. Weighted multivariate linear regression models were employed to examine associations between endometriosis diagnosis age and dietary theobromine intake. Subgroup categories and sample sizes are comprehensively summarized in Table 1. Model 1: adjusted for sociodemographic covariates (age, race/ethnicity, education level, family income, and BMI). Model 2: further adjusted for nutrient covariates (calorie, carbohydrate, and total fat consumption). Model 3: fully adjusted model, incorporating all model 2 variables plus smoking status, physical activity level, and dietary supplement use. To evaluate subgroup effects, stratified multivariate regression analysis were performed, controlling for all were performed except the stratification variable. Heterogeneity in associations across subgroups was assessed using the log-likelihood ratio test for interaction. Interaction effects were quantified by comparing −2 log – likelihood values between nested models with/without interaction terms. Statistical analyses were performed using: The R survey package (version 4.1-1); Free Statistics software (version 1.7.1; Beijing FreeClinical Medical Technology Co., Ltd., Beijing, China); R software (version 4.2.1; R Foundation for Statistical Computing, Vienna, Austria).^[21]^ MR analysis was conducted using R statistical software with the TwoSampleMR package.^[22]^ *F*-statistics were computed to evaluate the strength of each instrumental variable. In the MR analyses, the β coefficient was defined as the change in log odds ratio (log OR) of endometriosis per 1 − standard deviation increase in genetically predicted theobromine levels. The inverse variance weighted (IVW) method served as the primary approach for assessing associations between dietary theobromine and endometriosis risk. Complementary methods – weighted median, weighted-Egger, and weighted-mode – were additionally employed to validate IVW results. The advantages and limitations of these approaches have been previously described in the literature.^[23]^ Potential heterogeneity and directional pleiotropy were independently evaluated using the Cochran’s *Q* test and the MR-Egger intercept test, respectively. To examine the heterogeneity across single – nucleotide polymorphisms (SNPs), the Cochran’s *Q* statistic was utilized. A *P*-value exceeding .05 from this test suggests that the effects of SNPs are consistent. Regarding the assessment of directional pleiotropy, the MR-Egger intercept test was applied. A *P*-value > .05 from this test indicates the absence of significant bias.^[24]^ A leave-one-out sensitivity analysis was further performed to evaluate robustness. Statistical significance was defined as a 2-tailed *P*-value < .05.

**Table 1:** Characteristic of participants in the study, weighted.

Characteristics		Tertiles of dietary theobromine intake (mg/d)		
	Total	Low (0–8.15)	High (8.16–872)	*P*
	(n = 244)	(n = 122)	(n = 122)	
Age (yr)				
<30	24.00 (9.84)	12.00 (9.84)	12.00 (9.84)	.9608
30–40	76.00 (31.15)	37.00 (30.33)	39.00 (31.97)	
>40	144.00 (59.02)	73.00 (59.84)	71.00 (58.20)	
BMI (Kg/m^2^)				
<25	88.00 (36.07)	39.00 (31.97)	49.00 (40.16)	.3516
25–30	71.00 (29.10)	36.00 (29.51)	35.00 (28.69)	
>30	85.00 (34.84)	47.00 (38.52)	38.00 (31.15)	
Family income, n (%)				
Low	50.00 (21.37)	26.00 (22.41)	24.00 (20.34)	.8977
Medium	80.00 (34.19)	40.00 (34.48)	40.00 (33.90)	
High	104.00 (44.44)	50.00 (43.10)	54.00 (45.76)	
Race/ethnicity, n (%)				
Mexican American	24.00 (9.84)	15.00 (12.30)	9.00 (7.38)	.2768
Other race	10.00 (4.10)	4.00 (3.28)	6.00 (4.92)	
Non-Hispanic White	174.00 (71.31)	81.00 (66.39)	93.00 (76.23)	
Non-Hispanic Black	35.00 (14.34)	21.00 (17.21)	14.00 (11.48)	
Education level (yr)				
<9	3.00 (1.23)	3.00 (2.46)	0.00 (0.00)	.1018
9–12	30.00 (12.30)	18.00 (14.75)	12.00 (9.84)	
>12	211.00 (86.48)	101.00 (82.79)	110.00 (90.16)	
Marital status, n (%)				
Living with a partner	160.00 (65.57)	83.00 (68.03)	77.00 (63.11)	.1891
Living alone	81.00 (33.20)	39.00 (31.97)	42.00 (34.43)	
Smoking status, n (%)				
Yes	126.00 (51.64)	61.00 (50.00)	65.00 (53.28)	.6096
No	118.00 (48.36)	61.00 (50.00)	57.00 (46.72)	
Vigorous activity, n (%)				
Yes	78.00 (31.97)	35.00 (28.69)	43.00 (35.25)	.5436
No	158.00 (64.75)	83.00 (68.03)	75.00 (61.48)	
Unable to do activity	8.00 (3.28)	4.00 (3.28)	4.00 (3.28)	
Moderate activity, n (%)				
Yes	133.00 (54.51)	56.00 (45.90)	77.00 (63.11)	.027
No	104.00 (42.62)	62.00 (50.82)	42.00 (34.43)	
Unable to do activity	7.00 (2.87)	4.00 (3.28)	3.00 (2.46)	
Dietary supplements taken, n (%)				
Yes	143.00 (58.61)	73.00 (59.84)	70.00 (57.38)	.6975
No	101.00 (41.39)	49.00 (40.16)	52.00 (42.62)	
Calorie consumption (kcal/d), mean (SD)	1852.357 (760.874)	1717.026 (703.347)	1987.688 (794.365)	.0051
Protein consumption (g/d), mean (SD)	64.857 (29.309)	62.032 (29.183)	67.682 (29.281)	.1317
Carbohydrate consumption (g/d), mean (SD)	235.285 (113.074)	207.362 (98.420)	263.207 (120.076)	.0001
Total sugars consumptions (g/d), Median (IQR)	105.810 [64.015, 151.480]	73.780 (51.760, 129.280)	126.580 (81.995, 170.955)	<.0001
Total fat consumption (g/d), Median (IQR)	64.090 [43.950, 90.970]	60.800 (41.940, 89.510)	64.530 (46.650, 93.795)	.2606

BMI = body mass index, SD = standard deviation.

## 3. Results

### 3.1. Population characteristics of NHANES

The initial dataset included 275 female participants. After excluding those with missing endometriosis-related data and individuals outside the 20 to 54 age range, an additional 27 participants were excluded due to pregnancy, and 4 more due to theobromine deficiency, resulting in a final analytical sample of 244 participants.

Baseline characteristics of the 244 participants are summarized in Table 1. Among them, 126 participants (51.64%) reported being smokers, 143 participants (58.61%) reported using dietary supplements. Age distribution: 24 participants (9.84%) were aged < 30 years, 76 (31.15%) were 30 to 40 years, and 144 (59.02%) were >40 years. No significant differences were observed between the 2 groups in age, BMI, family income, race, education level, marital status, and smoking status (*P* > .05). Notably, participants in the higher theobromine intake group had a significantly higher prevalence of moderate physical activity (*P* = .027). Additionally, the higher intake group exhibited significantly greater daily calorie, carbohydrate, and total sugar intake compared to the lower intake group. These baseline characteristics not only reflect the demographic and behavioral diversity of the study population but also provide a foundational framework for subsequent analyses examining associations between these factors and endometriosis.

### 3.2. Relationship between dietary theobromine intake and age at endometriosis diagnosis

To investigate the association between dietary theobromine intake and endometriosis diagnosis age, 3 weighted multiple regression models were constructed: model 1: adjusted for sociodemographic variables, including age, race/ethnicity, education level, family income, marital status, and BMI. Model 2: further incorporated nutrient factors, such as calorie, carbohydrate, protein, and total fat consumption. Model 3 (fully adjusted): additionally controlled for lifestyle factors, including smoking status, vigorous activity, moderate activity, and dietary supplement use. Across all models, a consistent positive association was observed between endometriosis diagnosis age and dietary theobromine intake: model 1: β = 0.84, 95% CI [0.00, 1.67], *P* = .049; model 2: β = 1.40, 95% CI [0.54, 2.27], *P* = .02; model 3: β = 1.49, 95% CI [0.02, 2.95], *P* = .048. These results indicate that, in the fully adjusted model (model 3), each 1 mg increase in dietary theobromine intake may be associated with a 1.49-year delay in the age at endometriosis diagnosis (Table 2). Interaction tests confirmed no significant effect modification by age, BMI, or income strata (all *P*-interaction > .05 via log-likelihood ratio test).

**Table 2:** Association between dietary theobromine intake and the age at endometriosis diagnosis in the NHANES 1999–2004 cycles, weighted.

	No.	Crude β (95% CI)	*P*-value	Model 1* β (95% CI)	*P*-value	Model 2† β (95% CI)	*P*-value	Model 3‡ β (95% CI)	*P*-value
Dietary theobromine intake (mg/d)	244	1.08 (0.18–1.97)	.002	0.84 (0.00–1.67)	.049	1.4 (0.54–2.27)	.002	1.49 (0.02–2.95)	.048

BMI = body mass index, CI = confidence interval, NHANES = National Health and Nutrition Examination Survey.

* Model 1 adjusted for age, race/ethnicity, education level year, family income, marital status and BMI.

† Model 2 was adjusted for model 1 + calorie consumption, carbohydrate consumption, protein consumption and total fat consumption.

‡ Model 3 was adjusted for model 2 + smoking status, vigorous activity, moderate activity and dietary supplements taken.

### 3.3. MR of theobromine metabolites and endometriosis risk

To assess the causal relationship between theobromine metabolites and endometriosis, multiple MR methods were employed: MR-Egger regression, weighted median, simple mode, and IVW. The IVW method, the primary analytical approach, yielded an effect estimate of −0.1057 (95% CI: −0.2098 to −0.0016, *P* = .045), indicating a modest negative association between the exposure and the outcome. MR-Egger regression analysis was performed to evaluate potential horizontal pleiotropy. The MR-Egger intercept was −0.0265, with a *P*-value of .1533, suggesting no significant evidence of horizontal pleiotropy, which strengthens the reliability of the MR results. Collectively, these findings support the robustness of the MR analysis, with no major biases detected (Table 3).

**Table 3:** Causal effects of theobromine on endometriosis.

Exposure	Outcome	Method	nSNP	β	se	*P*
Theobromine	Endometriosis	MR-Egger	8	0.114600343	0.144736434	.458635231
Theobromine	Endometriosis	Weighted median	8	-0.03801081	0.069931957	.586758111
Theobromine	Endometriosis	Inverse variance weighted	8	−0.10572315	0.052640625	.044601363
Theobromine	Endometriosis	Simple mode	8	−0.00151478	0.114043727	.989773112
Theobromine	Endometriosis	Weighted-mode	8	−0.00407033	0.126795056	.975287146

se = standard deviation, SNP = single-nucleotide polymorphism.

### 3.4. Horizontal pleiotropy assessment and sensitivity analysis

The MR-Egger regression intercept showed no significant deviation from 0, indicating no evidence of horizontal pleiotropy (Table S1, Supplemental Digital Content, https://links.lww.com/MD/Q743). Heterogeneity tests further confirmed consistency across SNP effects: For the MR-Egger method, the *Q* statistic was 2.53 (*P* = .87).

For the IVW method, the *Q* statistic was 5.20 (*P* = .64). Both *P*-values exceeded .05, suggesting consistent effects of the SNPs and validating their use as instrumental variables for reliable causal inference (Table S2, Supplemental Digital Content, https://links.lww.com/MD/Q743). A leave-one-out sensitivity analysis was performed to assess the robustness of the findings, as excluding individual SNPs did not significantly alter the overall effect estimate. Most *P*-values remained below .05, indicating that the causal effect remained statistically significant regardless of the exclusion of any single SNP. This consistency underscores the stability and reliability of the causal effect estimate (Table S3, Supplemental Digital Content, https://links.lww.com/MD/Q743).

## 4. Discussion

To the best of our knowledge, this is the first study to integrate MR analysis with large-scale observational data from the NHANES cohort to comprehensively investigate the relationship between dietary theobromine intake and endometriosis risk. Our research specifically explored the potential causal association between theobromine intake and endometriosis development. Both cross-sectional analysis and MR results provided evidence supporting a protective association: Data from the NHANES cohort revealed that higher dietary theobromine intake was associated with an older age at endometriosis diagnosis. Specifically, our findings indicate that each 1-μg increase in dietary theobromine intake was associated with an approximately 1.49-year delay in the age at diagnosis. This observation indirectly implies that the onset age of endometriosis may also be delayed by approximately 1.49 years. MR analysis demonstrated an inverse association between theobromine levels and endometriosis risk (β = −0.1057, 95% CI: −0.2098 to −0.0016, *P* = .045), indicating that increased dietary intake of theobromine may be modestly associated with reduced endometriosis risk, though the effect size is limited and confidence intervals include near-null values. These findings were consistent across various sensitivity analyses, reinforcing the robustness of the protective effect of theobromine. Endometriosis is strongly associated with oxidative stress and inflammation. Studies indicate that patients with endometriosis exhibit elevated levels of both localized and systemic inflammation.^[25]^ This abnormal inflammatory state, driven by mechanisms such as immune angiogenesis and immune-endocrine interactions, may influence the development of endometriosis lesions and disease progression.^[1,25]^ Mechanistically, 1 proposed pathway involves inflammatory cytokines produced by lesion-resident immune cells or endometrial cells. These cytokines impair the immune system’s ability to clear aberrant endometrial cells, thereby facilitating lesion formation and growth. Additionally, inflammation may reduce endometrial cell apoptosis, promote cell proliferation, and enhance attachment to mesothelial cells by creating a pro-invasive microenvironment.^[26]^ Genetic evidence further supports this link: genes related to inflammation, immune response, angiogenesis, and steroid responsiveness (including those associated with progesterone resistance) show differential expression patterns in the eutopic endometrium of endometriosis patients.^[27]^ In the peritoneal microenvironment, menstrual reflux introduces high levels of immune cells, proteases, and pro-inflammatory cytokines, which may alter local conditions. It is hypothesized that endometrial stem/progenitor cells survive this environment, disseminate to the peritoneum, and contribute to lesion formation.^[28]^ Theobromine, a methylxanthine compound, exerts diverse biological activities with demonstrated health benefits, including protective effects against respiratory and cardiovascular diseases, cancer, obesity, diabetes, infertility, and neurological disorders.^[9,29]^ Central to these benefits are its anti-inflammatory and antioxidant qualities, which modulate the immune system by promoting the synthesis of anti-inflammatory mediators. This immunomodulatory effect helps preserve epithelial barrier integrity, thereby reducing the risk of inflammatory conditions such as dermatitis, intestinal inflammation, and osteoarthritis.^[12]^ Mechanistic studies have elucidated specific pathways underlying its anti-inflammatory actions: A study by Lee, HW et al demonstrated that theobromine activates the p38, JNK, and NF-κB signaling pathways in macrophages, leading to enhanced production of inflammatory mediators. This activation upregulates inducible nitric oxide synthase expression, increases nitric oxide (NO) levels, and promotes cyclooxygenase-2 (COX-2) expression, collectively elevating prostaglandin E2 production.^[30]^ Additionally, research in chondrocytes revealed that theobromine mitigates interleukin-1β (IL-1β) mediated inflammation primarily by reducing matrix metalloproteinases (MMPs), thereby dampening inflammatory responses.^[31]^ Another study discovered that a theobromine-containing diet for 1 week changed the makeup of lymphocytes in the spleen, mesenteric lymph nodes, and thymus, as well as the systemic and intestinal IgG concentrations in young, healthy Lewis rats.^[32]^ These findings lay the groundwork for understanding how theobromine could be used therapeutically for inflammatory-related diseases. This study exhibits several strengths. First, it may be the first to explore the association between the age at endometriosis diagnosis and theobromine intake, thereby laying a foundation for future research in this area. Second, by integrating 2-sample MR analysis with NHANES cohort data, the study not only provides a comprehensive interpretation of the cross-sectional findings but also establishes a causal relationship between theobromine levels and endometriosis. Additionally, sensitivity analyses consistently supported the association between dietary theobromine intake and the age at endometriosis diagnosis, validating the reliability of the data. Finally, the use of a representative sample enhances the generalizability of the results to the adult population in the United States.

However, the study is not without limitations. Firstly, although the integration of 2-sample MR analysis with NHANES data aids in establish a causal relationship between theobromine levels and endometriosis, the cross-sectional design of the NHANES data restricts the ability to draw definitive causal conclusions regarding dietary theobromine intake and the age at endometriosis diagnosis; thus, further longitudinal studies are required to confirm causality. Secondly, reliance on 24-hour dietary recall interviews may introduce recall bias. Thirdly, unmeasured confounding factors, such as genetic influences on the age of endometriosis onset, could have affected the results. Additionally, while the MR analysis achieved formal statistical significance (*P* = .045), the borderline *P*-value and modest effect size (β = −0.1057, equivalent to OR ≈ 0.90) indicate that the observed protective association requires validation in larger studies. Lastly, the use of NHANES data restricts the generalizability of findings to the American population, and the study’s focus on individuals aged 20 to 54 may introduce selection bias. Future research should investigate whether these findings apply to other countries and age groups.

## 5. Conclusion

This study provides suggestive evidence for a potential protective effect of dietary theobromine intake against endometriosis. The modest effect size (β = −0.1057) and borderline significance (*P* = .045) in MR analysis warrant cautious interpretation and replication. Both cross-sectional NHANES data and MR analysis consistently indicated that higher theobromine intake correlates with a later age of diagnosis and a potentially reduced risk of endometriosis. Specifically, each 1-microgram increase in theobromine intake was associated with an approximate 1.49-year delay in diagnosis age. These findings underscore the potential value of dietary interventions in managing and preventing endometriosis. Further research should explore the underlying mechanisms and investigate theobromine as a possible preventive measure for the disease.

## Acknowledgments

The authors thank the investigators of the GWAS publications on theobromine and endometriosis as well as the FinnGen study for making their data publicly available. We also acknowledge the National Health and Nutrition Examination Survey (NHANES) for providing the essential data for this research.

## Author contributions

**Conceptualization:** Dongmei Huang, Yingqin Huang.

**Data curation:** Feng Liang.

**Investigation:** Feng Liang.

**Methodology:** Baoli Xie.

**Resources:** Feng Liang.

**Supervision:** Dongmei Huang, Feng Liang.

**Validation:** Dongmei Huang, Baoli Xie.

**Writing – original draft:** Yingqin Huang, Feng Liang, Baoli Xie.

**Writing – review & editing:** Dongmei Huang, Yingqin Huang.

## Supplementary Material


