# Supplementary material for: Exploring the impact of dietary theobromine on endometriosis risk: Evidence from Mendelian randomization and NHANES data
Source: Medicine (Baltimore). 2025 Nov 21;104(47):e45989. doi: 10.1097/MD.0000000000045989 (PMC12643750; doi:10.1097/MD.0000000000045989)
Supplement: Supplementary file 1 [file medi-104-e45989-s001.pdf]

Table S1. pleiotropy_results

exposure	outcome	egger_intercept	se	p
Theobromine	Endometriosis	-0.027	0.02	0.15

Table S2. heterogeneity_results

exposure	outcome	method	Q	Q_pval
theobromine	endometriosis	MR Egger	2.53	0.87
theobromine	endometriosis	Inverse variance weighted	5.2	0.64

Table S3. leave_one_out_results

exposure	outcome	SNP	b	se	p
theobromine	endometriosis	rs117824460	-0.13	0.06	0.03
theobromine	endometriosis	rs1801272	-0.12	0.06	0.03
theobromine	endometriosis	rs2316205	-0.09	0.07	0.10
theobromine	endometriosis	rs2523264	-0.11	0.06	0.04
theobromine	endometriosis	rs2529273	-0.12	0.06	0.04
theobromine	endometriosis	rs35755165	-0.09	0.06	0.10
theobromine	endometriosis	rs56113850	-0.07	0.06	0.26
theobromine	endometriosis	rs67210567	-0.12	0.06	0.03
theobromine	endometriosis	All	-0.11	0.05	0.04
